# Numerical Study of Melt-Spinning Dynamic Parameters and Microstructure Development with Ongoing Crystallization

**DOI:** 10.3390/polym16172398

**Published:** 2024-08-23

**Authors:** Xiangqian Liu, Pei Feng, Chongchang Yang, Zexu Hu

**Affiliations:** 1 College of Mechanical Engineering, Donghua University, Shanghai 201620, China; lxq_study163@163.com (X.L.); ycc@dhu.edu.cn (C.Y.); huzexu@dhu.edu.cn (Z.H.); 2 Engineering Research Center of Advanced Textile Machinery, Donghua University, Shanghai 201620, China; 3 State Key Laboratory of Fiber Material Modification, Donghua University, Shanghai 201620, China

**Keywords:** melt spinning, structure, dynamics, two-phase model, crystallization

## Abstract

In response to an investigation on the paths of changes in the crystallization and radial differences during the forming process of nascent fibers, in this study, we conducted numerical simulation and analyzed the changes in crystallization mechanical parameters and tensile properties through a fluid dynamics two-phase model. The model was based on the melt-spinning method focusing on melt spinning, the environment of POLYFLOW, and the method of joint simulation, coupled with Nakamura crystallization kinetics, including the development of process collaborative parameters, stretch-induced crystallization, viscoelasticity, filament cooling, gravity term, inertia, and air resistance. Finally, for nylon 6 BHS and CN9987 resin spinning, the model successfully predicted the distribution changes in temperature, velocity, strain rate tensor, birefringence, and stress tensor along the axial and radial fibers and obtained the variation pattern of fibers’ crystallinity along the entire spinning process under different stretching rates. Furthermore, we also explored the effects of spinning conditions, including inlet flow rate, winding speeds, and the extrusion temperature, on the fibers’ crystallization process and obtained the influence rules of different spinning conditions on fiber crystallization. Knowing the paths of changes in mechanical performance can provide important guidance and optimization strategies for the future industrial preparation of high-performance fibers.

## 1. Introduction

Melt spinning is a key technology, widely used in the industrial manufacturing of synthetic fibers. Due to the many limitations of existing technology, significant improvements in fibers leading to higher performance are constrained. In long spinning, the process of interaction between the parameters that affect the changes in the fiber structure and performance is far from adjusting a single factor that could allow for the optimization of fiber structure and performance [1,2,3]. The collaborative regulation of multiple factors brings an enormous workload to experiments, and experimental results are often limited to specific systems, with poor scalability [4,5,6]. The established online detection methods are insufficient in detecting the variation path of fibers’ microscopic parameters throughout the spinning process; however, this is crucial for understanding and predicting the formation of non-equilibrium fibers’ structures during the spinning process.

Numerical simulation is an important tool that can provide insights and predictions of the fiber structure during the formation process at the microscale, as well as motion state information, such as the velocity field, temperature field, and stress field [7,8,9]. To theoretically understand the mechanism of fiber formation during the spinning process and compensate for the shortcomings of experimental methods, Keunings et al. [10] modeled and simulated a three-dimensional spinning process of UCM fluids with the method of finite elements. The work simulated polymer melt as the isothermal Newtonian fluid in their modeling method. However, taking into account crystallization as a process of heat as well as energy transformation, the significant drawback of isothermal Newtonian fluid is that it cannot handle strain changes related to nonlinear viscoelastic effects. Furthermore, it cannot reflect the deformation of fibers during extrusion, which is inconsistent with reality. Huynh and Tanner [11] simulated and analyzed the non-isothermal spinning process of Newtonian fluids. The research results showed that there was a significant temperature difference in the radial direction of fibers, and the above simulation analysis did not consider the crystallization phase transition process of polymer materials. In fact, the equation used in the model was the Maxwell constitutive equation, which reflected the viscoelastic properties of fluids and affected the crystallization of fiber formation at length. This equation has the disadvantage of being a single-phase model and cannot reflect the formation of the neck (for example, for a winding speed of above 2000 m/min, their model’s reflected speed will not reach the platform zone). In Wilkie A’s study [12], there was a significant temperature deviation in the radial direction of cooling fibers, with the fibers’ central temperature higher than the surface temperature. Although they simulated the amorphous and semi-crystalline phases as different constitutive models, they ignored the effects of fiber cooling, air resistance, and, in some cases, inertial forces on boundary conditions, free surfaces, and phase transitions when establishing the model. Thus, the robustness of the model did not match the actual winding speed and flow rate of spinning. A similar viewpoint was also expressed in Tae Hwan Oh’s study [13], in which the fibers’ temperature was non-uniformly distributed on the cross-section.

These researchers used the average temperature of the cross-section instead of the fibers’ surface temperature, which can lead to an overestimation of thermal conductivity with the surrounding environment, resulting in incorrectly calculated temperature fields. Due to the fact that stress is a function of temperature in the flow of viscoelastic fluids, deviations in temperature field calculations can cause certain deviations in the calculated stress, leading to incorrect molecular orientation and crystallinity, ultimately affecting the evaluation of the fiber structure and properties. Sun and Wang et al. [14,15] considered the phenomenon of flow-induced the crystallization and established a simulation of two-dimensional viscoelastic spinning. The model predicted extrusion swelling, temperature, and birefringence data at the spinneret, which was consistent with experiments. However, the established model was essentially a single-phase model, which could not describe the radial structure and crystal changes of fibers. They essentially did not append different constitutive equations for the crystalline and amorphous phases, which, in a sense, limited their understanding of the complex phenomena that occur during high-melt-spinning processes. Moreover, the fibers’ solidification point is not naturally generated in the model formula but, rather, a transition point after reaching the polymer glass transition temperature by setting a fixed temperature.

In this study, based on the environment of development regarding POLYFLOW [16,17], a method of joint simulation was used to couple the Nakamura crystallization kinetics equation, and a two-phase mathematical model with a process of synergistic parameters including stretch induced the crystallization, viscoelasticity, wire cooling, gravity term, inertia, and air resistance. The model can predict the changes in crystal mechanical parameters, tensile properties, and microcrystalline crystallinity of fibers during the process of fiber formation, including radial changes in fiber temperature, velocity gradient, birefringence index, strain rate tensor, and stress tensor distribution, along the axial and radial directions, as well as the effect of different stretching on fiber crystallization. In addition, the influence of spinning conditions, including the inlet flow rate, winding speed, and extrusion temperature, on the changes in the fiber crystallization process was explored through the model. Monitoring the paths of changes in these process parameters is crucial for the industrial preparation of high-performance fibers.

## 2. Mathematical Model

### 2.1. Method

**Figure 1:**
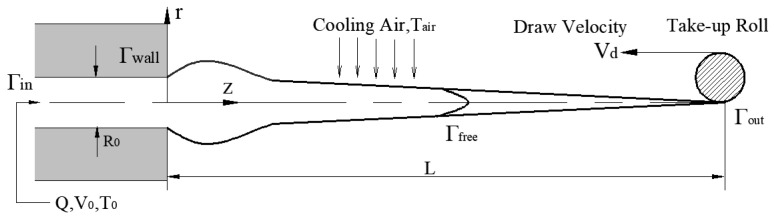
Process of melt spinning fiber (the computational domain covers the entire enclosed area from the fluid to the inlet of the spinneret. $\Gamma_{in}$ is the inlet boundary of the fluid, $\Gamma_{\mathrm{wall}}$ is the wall surface of the spinneret, $\Gamma_{\mathrm{out}}$ is the outlet of the fluid, $\Gamma_{\mathrm{free}}$ is the free surface of the fiber).

The problem of flow considered in our study is the steady-state flow of fiber materials undergoing phase transformation and crystallization during the process of melt spinning [18,19]. In the cylindrical coordinate system, it was assumed that the flow field was axisymmetric, as shown in Figure 1. The radius of the spinneret hole is *r*_0_, the outlet speed is V_0_, the temperature is *T*_0_, the crystallinity is θ, *L* is the distance of the filament along the process of spinning, and *V*_d_ is the stretching speed of the spinning. For the incompressible steady-state, non-isothermal viscoelastic fluids, the process of fiber-forming phase transition involves two dynamic processes: the formation and growth of crystal nuclei, which are processes of energy conversion [20,21]. At the same time, the establishment of the following models is based on incompressible viscoelastic fluids.

As shown in Figure 1, the construction of the model was based on a cylindrical coordinate system, and the origin was selected at a position with uniform radial and axial velocities. For fluids in an incompressible stable flow state, the steady-state equation of melt spinning can be solved through the three major equilibrium equations of the continuity, momentum, and energy. In the radial transmission, assuming that the axial velocity and temperature were uniform on the cross-section, and the flow was locally uniformly uniaxial extended, the expressions of the three major equations can be expressed as Equations (1)–(3).

#### 2.1.1. Mass Balance

(1) $$w=\rho \frac{\pi {D_{0}}^{2}}{4}v_{z}$$

where w is the polymer quality, ρ is the density, $D_{0}$ is the fiber diameter, and $V_{0}$ is the velocity component of the fibers along the z direction.

#### 2.1.2. Momentum Balance


(2) $$w\frac{dv_{z}}{dz}=\frac{d}{dz}[A(\tau_{zz}-\tau_{rr})]-\pi B\mu_{0}(v_{z}-v_{d})+\rho gA+\frac{\pi s}{2}∙\frac{dD_{0}}{dz}$$

In Equation (2), the first term is the inertia term, the second term is the air resistance term, and *B* is the Bingham number, which represents the resistance of cooling air per unit length of fiber. $v_{d}$ is the velocity component of the blowing air, $\mu_{0}$ is the viscosity value of the blowing air, *g* is the gravitational acceleration, *A* is the cross-sectional area of the fibers, and *S* is the total surface tension acting on the filament. This equation is not directly related to the constitutive equation of the amorphous and semi-crystalline phases of fibers.

#### 2.1.3. Energy Conversion

(3) $$\rho c_{p}v_{z}\frac{dT}{dz}=(\tau_{zz}-\tau_{rr})\frac{dv_{z}}{dz}-\frac{4h(T-T_{0})}{D_{0}}+\rho \Delta h_{f}v_{z}\frac{d\theta_{\mathrm{av}}}{dz}$$

where $C_{p}$ is the specific heat capacity, and it is related to the spinning temperature and crystallinity. The first term in Equation (3) represents viscous dissipation, the second term represents convective heat transfer between fibers and air, *h* is the coefficient of thermal convective heat transfer, and the third term represents the latent heat released due to the crystallization of fibers. $\Delta h_{f}$ is the crystallization heat per unit mass, and $\theta_{\mathrm{av}}$ is the average crystallinity of the entire spinning system. In establishing the energy equation, Denn et al. [22] neglected the radiation effect and the axial conductivity of heat while considering that the crystallization of fibers is an exceptional process. The last term in the energy equation retained a term of heat released by late heat.

#### 2.1.4. Tensile Kinematics and Stress Field

Assuming that the shear stress generated in the spinneret was already in a relaxed state, and the flow field during the process of spinning was a locally uniform uniaxial tensile flow, relevant researchers have adopted this assumption during melt spinning [23,24,25]. Therefore, under the condition of constant density, the velocity tensor can be expressed as in Equation (4).

(4) $$\nabla v=\left[\begin{array}{ccc} \frac{dv_{z}}{dz} & 0 & 0\\ 0 & -\frac{1}{2}\frac{dv_{z}}{dz} & 0\\ 0 & 0 & -\frac{1}{2}\frac{dv_{z}}{dz} \end{array}\right]$$

The stress tensor can be written as Equation (5).

(5) τ=τzz   τrr   τθθ

where zz, *rr* and θθ represent stress components in three different directions.

#### 2.1.5. Constitutive Model

Based on the process of spinning design and the characteristics of the materials, the characteristics of the constitutive equations of the Maxwell model, Giesekus model, and Phan Thien Tanner (PTT) were compared. The expression of control equations for the three constitutive models is (6–18).

Maxwell model:

(6) $$\sigma +\lambda [\frac{\partial \sigma }{\partial t}+v∙\nabla \sigma -(\nabla v)∙\sigma -\sigma ∙{\left(\nabla v\right)}^{T}-\sigma ∙(v\nabla \ln T)]=\mu [\nabla v+{\left(\nabla v\right)}^{T}]$$

where σ is the stress tensor, μ is the shear viscosity, *t* is the melting time, and λ is the relaxation time.

For the flow of incompressible steady-state fluid, the above equations can be written as a component form with the following expressions (7) and (8) during simulation [13].

(7) $$\tau_{\mathrm{zz}}+\lambda [v_{z}\frac{d\tau_{zz}}{dz}-2\tau_{zz}\frac{dv_{z}}{dz}-v_{z}\tau_{zz}\frac{d\ln T}{dz}]=2\mu \frac{dv_{z}}{dz}$$

(8) $$\tau_{\mathrm{rr}}+\lambda [v_{z}\frac{d\tau_{rr}}{dz}-2\tau_{rr}\frac{dv_{z}}{dz}-v_{z}\tau_{rr}\frac{d\ln T}{dz}]=2\mu \frac{dv_{z}}{dz}$$

where $\tau_{\mathrm{zz}}$ is the axial force of the fiber, and $\tau_{\mathrm{rr}}$ is the radial force of the fiber.

The mathematical relationship among σ and $\tau_{\mathrm{zz}}$, $\tau_{\mathrm{rr}}$ is Equation (9).

(9) $$\sigma =\tau_{\mathrm{zz}}-\tau_{rr}$$

Giesekus model:

(10) $$\tau =\sum_{t}^{n}\tau_{j}$$

(11) $$\frac{\partial \tau_{j}}{\partial t}+v∙\nabla \tau_{j}-\nabla v^{T}∙\tau_{j}-\tau_{j}∙\nabla v+\frac{1}{\lambda_{j}}(\tau_{j}+\frac{\psi \lambda_{j}}{\eta_{j}}\tau_{j})=\frac{\lambda_{j}(\nabla v+\nabla v^{T})}{\eta_{j}}$$

where $\lambda_{j}$ and $\eta_{j}$ are the relaxation time and shear viscosity under the j model; ψ is the liquidity factor, with a value range of 0–1.

PTT model:

(12) $$\exp[\frac{\varepsilon \lambda }{\eta }\mathrm{tr}(\tau )]\tau +\lambda [(1-\frac{\delta }{2})\overset{\nabla }{\tau }+\frac{\delta }{2}\overset{\Delta }{\tau }]=2\eta D$$

wherein

(13) $$\overset{\nabla }{\tau }=v∙\nabla \tau -\nabla v^{T}∙\tau -\tau ∙\nabla v$$

and

(14) $$\overset{\Delta }{\tau }=v∙\nabla \tau +\tau ∙\nabla v^{T}+\nabla v∙z$$

The above equations, Equations (13) and (14), represent the upper- and lower-body derivatives of the polymer stress tensor τ under the condition of a steady state, respectively. δ is the slip factor between the continuous medium and the molecular network model, ε represents the change in tensile viscosity, λ is the relaxation time, and *D* is the strain rate tensor; its expression is shown in Equation (15).

(15) $$D=\frac{\left(\nabla v+\nabla v^{T}\right)}{2}$$

Assuming that the principle of “time temperature equivalence” conformed to the temperature-dependent relationship of the viscoelasticity of spinning materials, the relationship between viscosity η, relaxation time λ, and temperature *T* can be expressed as (16) and (17).

(16) $$\eta (T)=\alpha_{T}(T_{0})$$

(17) $$\lambda (T)=\alpha_{T}(T_{0})$$

where $T_{0}$ is the reference temperature, *T* is the absolute temperature, and $\alpha_{T}$ is the conversion factor; its value can be calculated using the Arrhenius equation, Equation (18).

(18) $$\ln(\alpha_{T})=\ln(\frac{\eta }{\eta_{0}})=\frac{\Delta H}{R}(\frac{1}{T}-\frac{1}{T_{0}})$$

where ΔH is the viscous activation energy of the material, and *R* is the gas constant at the reference temperature.

#### 2.1.6. Definition of Dimensionless Variables

To improve computational efficiency, the dimensionless variable expressions (19–31) were introduced.

Inertia:

(19) $$D_{1}=\frac{\rho {v_{0}}^{2}}{G}$$

Air drag:

(20) $$D_{2}=\frac{\pi \eta LB\rho {v_{0}}^{2}}{GW}$$

Gravity:

(21) $$D_{3}=\frac{gL\rho }{G}$$

Surface tension:

(22) $$D_{4}=\frac{S}{2G}\sqrt{\frac{\pi \rho v_{0}}{W}}$$

Heat convection:

(23) $$D_{5}=\frac{2Lh}{c_{p}}\sqrt{\frac{\pi }{\rho v_{0}W}}$$

Viscous dissipation:

(24) $$D_{6}=\frac{G}{\rho c_{p}T_{0}}$$

Latent heat of crystallization:

(25) $$D_{7}=\frac{\Delta H_{f}\theta_{\infty }}{c_{p}T_{0}}$$

Dimensionless axial distance:

(26) $$z^{*}=\frac{z}{L}$$

Dimensionless velocity:

(27) $${v_{z}}^{*}=\frac{v_{z}}{v_{0}}$$

Dimensionless temperature:

(28) $$T^{*}=\frac{T}{T_{0}}$$

Dimensionless extra stress tensor:

(29) $$\tau^{*}=\frac{\tau }{G}$$

Relative velocity:

(30) $$v_{r}=\frac{v_{d}}{v_{0}}$$

Deborah number of semi-crystalline phase:

(31) $$D_{\mathrm{esl}}=\frac{\lambda ∙v_{0}}{L}$$

#### 2.1.7. Evolution Methods

There are two types of variables in the model; one includes the macroscopic velocity change *V*, the temperature change *T*, and the fiber cross-sectional size change. Another type is the microstructural changes in total stress tensor *S*, strain rate tensor *D*, and birefringence Δn in the amorphous and semi-crystalline regions. Before beginning crystallization, the independent fluid variable is *V* as well as *T*. *D*_zz_ is relative to the axial direction of the fibers. *D*_rr_ is relative to the radial direction of the fibers.

Momentum balance:

Both sides of Equation (2) use $L/Wv_{0}$, which can eliminate the diameter $D_{0}$ of the filament, and, at this point, the momentum balance control equation will become Equation (32).

(32) $$D_{1}\frac{d{v_{z}}^{*}}{{d_{z}}^{*}}=\frac{d}{dz^{*}}[\frac{{\tau_{zz}}^{*}-{\tau_{rr}}^{*}}{{v_{z}}^{*}}]-D_{2}({v_{z}}^{*}-v_{r})+\frac{D_{3}}{{v_{z}}^{*}}-D_{4}{\left({v_{z}}^{*}\right)}^{-\frac{3}{2}}\frac{d{v_{z}}^{*}}{dz^{*}}$$

Energy equation:

Multiply both sides of Equation (3) by $LT_{0}/\rho c_{p}v_{0}$; thus, after sorting, Equation (33) can be obtained.

(33) $$\frac{dT^{*}}{dz^{*}}=-D_{5}(v_{z}^{*}{)}^{-\frac{1}{2}}(T^{*}-T_{0})+D_{6}\frac{{\tau_{zz}}^{*}-{\tau_{rr}}^{*}}{{v_{z}}^{*}}\frac{d{v_{z}}^{*}}{dz^{*}}+D_{7}\frac{1}{{v_{z}}^{*}}{\left[-\ln(1-x)\right]}^{\frac{m-1}{m}}(1-x)\exp(\mathrm{tr}\;\tau^{*})$$

The evolution equation for birefringence is as follows:

(34) $$\frac{d\Delta n^{*}}{dz^{*}}=(1-\theta )\frac{c_{op}\mu v_{0}}{L_{0}}({\tau_{zz}}^{*}-{\tau_{rr}}^{*})+\theta f_{c}\frac{d\Delta {n_{c}}^{*}}{dz^{*}}$$

where $f_{c}$ is the crystal orientation factor, $f_{c}=0.9$ [16], and $c_{op}$ is the optical coefficient of stress.

The evolution equation for $S_{zz}$ is as follows:

(35) $$\frac{{\mathrm{dS}}_{\mathrm{zz}}}{dz^{*}}=\frac{2S_{zz}}{{v_{z}}^{*}}\frac{d{v_{z}}^{*}}{dz^{*}}-\frac{\sigma S_{zz}}{{v_{z}}^{*}D_{esc}}+\frac{2}{3v_{z*}}\frac{d{v_{z}}^{*}}{dz^{*}}-\frac{1.7(1-w)}{{v_{z}}^{*}}(\frac{7}{15}+\frac{11}{14}S_{zz})\frac{d{v_{z}}^{*}}{dz^{*}}-\frac{3wS_{zz}}{{v_{z}}^{*}}(S_{zz}+0.34)\frac{{\mathrm{dv}}_{z}^{*}}{dz^{*}}$$

The $S_{\mathrm{rr}}$ and $S_{\theta \theta }$ components are eliminated using axial symmetry and the condition for *S* being traceless.

The expression before crystallization starts is (36).

(36) $$\tau^{*}=Ec^{*}-\delta$$

The expression after crystallization starts is (37).

(37) $$\tau^{*}=\frac{E}{1-x}c^{*}-\delta +3S+6D_{esc}{\left(\nabla^{*}v^{*}\right)}^{T}:<SSSS>$$

Considering the influence of the elastic modulus of the amorphous phase on the crystalline phase, the influence factor E/(1−x) is introduced in Equation (37).

Equation (38) can be obtained through the energy equation.

(38) $$\rho c_{p}(\frac{\partial T}{\partial t}+v_{z}\frac{\partial T}{\partial t})=\frac{1}{r}\frac{\partial }{\partial r}(r\lambda_{*}\frac{\partial T}{\partial r})+\eta_{0}r^{2}+Q$$

where ρ is the density, Cp is the specific heat capacity at time *t*, *t* is the time, $\eta_{0}$ is the kinematic viscosity, *T* is the spinning temperature, *r* is the axial radius, *z* is the axial length, $\lambda_{*}$ is the thermal conductivity, $v_{z}$ is the flow velocity along the *z* direction, and $Q^{*}$ is the release of heat per unit volume during the crystallization process, which is the latent heat term of crystallization.

Among them, the latent heat of the crystallization term $Q^{*}$ can be expressed by the crystallization kinetics expression (39) [26].

(39) $$Q^{*}=\rho \theta_{\infty }\Delta H_{C}\frac{d\theta }{dt}$$

where $\theta_{\infty }$ is the maximum crystallinity, and $\Delta H_{c}$ is the enthalpy of crystallization. It is not difficult to see that crystallization is not only affected by the extrusion temperature, cooling air temperature, and tensile stress but that these variables also change over time.

Norioki K et al. [27], H W Jing et al. [28], and Evans et al. [29] each derived the dependence of crystallinity θ on time from a statistical perspective, and their expression equations are shown in (40).

(40) $$\frac{d\theta }{dt}=nk_{s}(T,E(t))(1-\theta ){\left[-\ln(1-\theta )\right]}^{\frac{n-1}{n}}$$

Among them, *n* is the Avrami index, which is used to describe the mechanism and rate of crystal growth during the process of crystallization. Its value can be fitted by the Avrami equation experimental data. When the value of *n* is 1, it indicates that the crystal is growing linearly in one dimension. When the value of *n* is 2, it indicates that the crystal is growing in two dimensions. When the crystal is growing in three dimensions, the value of *n* is 3.

By using the iterative integration method on Equation (40) above, an integrated equation can be obtained, as shown in Equation (41).

(41) $$\theta =1-\exp[-(\int_{0}^{t}k_{s}(T,E(t)\mathrm{dt}){)}^{n}]$$

where $k_{s}(T,E(t))$ is the crystallization rate at time *t*.

For flow (shear)-induced crystallization, the rate of crystallization is controlled by Equation (42).

(42) $$k_{s}(T,\overset{∙}{\gamma }){=(\ln2)}^{\frac{1}{n}}(\frac{1}{\kappa_{t}})\exp(\frac{-\Delta H}{R(T-T_{\infty })})∙\exp(-\frac{k_{k}}{Tf(T_{m}(T,\overset{∙}{\gamma })-T)})$$

where $T_{\infty }$ represents the temperature at which the filament glass melts along the spinning process, $\kappa_{t}$ is the reference factor that affects crystallization, $\overset{∙}{\gamma }$ is the shear rate, $k_{k}$ is the nucleation index, $T_{m}$ is the melting point, and *R* is the gas constant.

The control equation for the crystallization coupled with Nakamura kinetics [30] induced by stretching (orientation) is (43).

(43) $$k_{s}(T,f_{ac})=k_{\max}\exp[-4\ln2\frac{T-T_{\max}}{D^{*}}+{\mathrm{cf}}_{ac}^{2}]$$

where $f_{ac}$ is the orientation factor, *c* is the stress-inducing factor, $D^{*}$ is the half-crystalline width, $K_{\max}$ is the maximum crystallization rate, and $T_{\max}$ is the temperature at the maximum rate of crystallization.

Almost all crystallization processes in chemical fiber technology are carried out in an oriented state [31]. Therefore, the rate of the crystallization kinetics model constructed in our models is based on the above equations.

wherein

(44) $$f_{ac}=\frac{\Delta \alpha }{\Delta \alpha_{i}}$$

Δα is the birefringence in the amorphous region, and the calculation equation is (44). $\Delta \alpha_{i}$ represents the inherent birefringence of the corresponding amorphous region. The amorphous birefringence is related to the laws of stress optics and the first normal force difference.

(45) $$\Delta \alpha =c_{op}^{*}\eta \frac{v_{0}}{L_{0}}(\tau_{zz}-\tau_{rr})$$

where $c_{op}^{*}$ is the stress optical coefficient, and $\tau_{zz}$ and $\tau_{\mathrm{rr}}$ represent the stress components along the *z* and *r* directions, respectively. The above three equations are the final kinetic models for evaluating crystallization.

Viscosity function:

For the flow of an incompressible polymer, the expression for its viscosity function can be obtained using the method of Sun et al. [14] as (46).

(46) $$\eta (T)=T∙\exp\langle \frac{\Delta H}{R}(\frac{1}{T}-1)\rangle$$

In the formula, *R* is the gas constant at the standard atmospheric pressure. Considering the influence of crystallinity on viscosity, the dependence of viscosity on crystallinity is further quantified by introducing Equation (47).

(47) $$\eta^{*}(T)=\eta (T)\exp\langle 4\theta^{2}\rangle$$

### 2.2. Control of Boundaries

$\Gamma_{in},\Gamma_{wall},\Gamma_{out}$, and $\Gamma_{free}$ represent the inlet boundary of the fluid, the wall of the spinneret, the outlet of the fluid, and the free surface of the fibers. The boundary condition for the spinning process was defined as (48–51), taking into account the full development of the fluid motion.

(48) $$\Gamma_{in}:\;v=v(r),\tau_{p}=\tau_{p}(r)$$

(49) $$\Gamma_{\mathrm{wall}}:\;v=0,T=T_{wall}$$

(50) $$\Gamma_{\mathrm{out}}:\;v_{z}=v,F_{r}=0,\nabla T∙n=0$$

(51) $$\Gamma_{free}:\;\sigma ∙n=0,v∙n=0,\nabla T∙n=-Bi(T-T_{0})$$

where σ is the total stress tensor and *Bi* is the Biot number; its calculation equation is shown in (52).

(52) $$Bi\equiv \frac{f_{h}∙L}{k}$$

where $f_{h}$ is the thermal conversion efficiency, calculated using the method of Kase and Matsuo [32] and expressed as (53).

(53) $$f_{h}=1.98A^{-0.333}v^{0.333}{\left[1+\frac{8v_{\mathrm{air}}}{v}\right]}^{0.167}$$

A is the cross-sectional area of the free surface of the fibers, and $v_{\mathrm{air}}$ is the speed of blowing air.

## 3. Numerical Method and Solution Procedure

**Figure 2:**
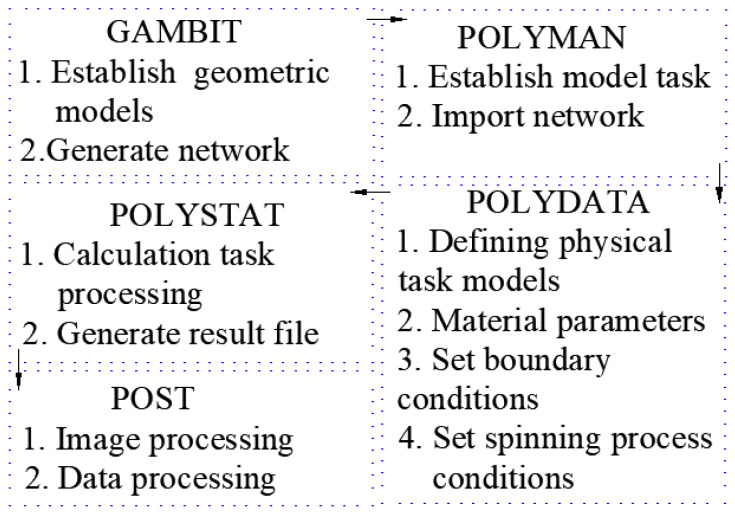
Block diagram of model solution.

During the simulation, the commercial software POLYFLOW was specifically used for solving non-Newtonian fluids. The model of crystallization kinetics was defined based on the transport characteristics of POLYFLOW and user-defined functions. The steps of the specific solution are shown in Figure 2. The hardware configuration environment used Windows 10 as the operating system, with an Inter (R) Core (TM) i9-10900, a CPU frequency of 208 GHz, and 32 GB of RAM on a PC. Considering the expansion of the fiber outlet and ensuring the accuracy of the calculation, mesh refinement was carried out at the inlet of fluid, followed by a gradually sparse mesh with a sparsity deviation of 2.05. In each unit, the velocity vector and stress component were solved using the DEVSS SU difference function [19].

The problem of convergence is the key to the success of simulation calculations. In our study, in order to improve the possibility of convergence and ensure the accuracy of simulation calculations, iterative functions and linear functions were used to solve the stress and strain rate tensors in the control equations. Considering that the deviatoric stress tensor is decomposed into elastic and viscous components using the EVSS (Elastic Viscous Splitting of the Stress) method, the SUPG method was advantageous for coupling between equations, with a numerical convergence standard of 10^−6^.

## 4. Numerical Simulations and Input Parameters

**Figure 3:**
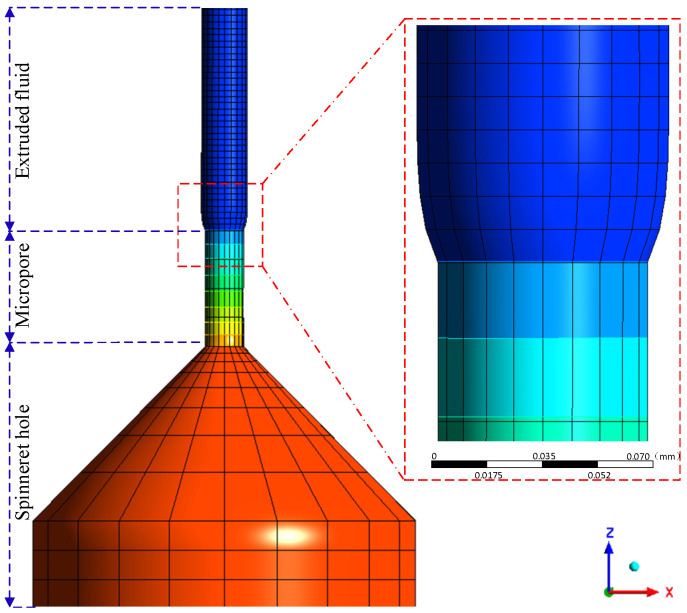
Model and grid division.

Selecting circular cross-section fibers as simulation objects, using Gambit to establish a geometric model of circular cross-section fibers, and meshing the geometric model, as shown in Figure 3, all meshes in the model geometry were tetrahedral structures. For the fluid inlet, surface mesh generation was used, and the free end was swept. To ensure the accuracy of simulation calculations, the “Implicit Euler” method was coupled with the “Picard Iterative” function. For the coupling calculation between free surface and fluid equations, in order to avoid the influence of free boundary repositioning on some grids, the “Upwinding in the kinematic equation” and “Optotimesh-3D” grid reset methods were adopted. The maximum ratio of aspect for grid partitioning was 3.328, the minimum unit mass was 0.021, the minimum orthogonal mass was 0.11, the maximum deviation was 0.102, and the shrinkage difference was 0.002. The total number of vertices in the meshing situation was 1035, the number of data segments was 1858, and the total number of planes was 824.

During the cooling process of fiber formation, when the surface temperature of the fibers is lower than the melting point temperature, the fibers will undergo crystallization, and different conditions of spinning and winding speeds will cause radial differences in the fibers and changes in the path of microcrystalline crystallization. Therefore, we simulated and analyzed mathematical models under the process of radius r_0_, spinning process *L*, and temperature *T* at the outlet of the spinneret.

### 4.1. The Influence of Different Constitutive Models on Extrusion

**Figure 4:**
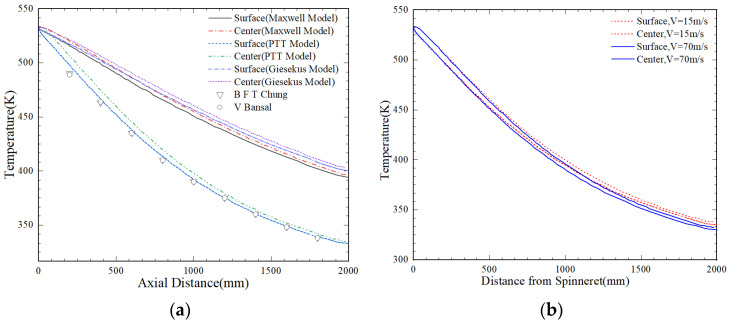
Temperature variation of (**a**) different constitutive equations; (**b**) different take-up speeds throughout the spinning process [35,36].

Three models, the Maxwell model, PTT model, and Giesekus model, were selected based on experience and the theory of fluid [33,34]. The Maxwell model uses two parameters, shear viscosity and relaxation, to represent the liner viscoelastic behavior of fluids. However, this model cannot accurately describe the viscoelastic properties of fluids during deformation and flow. The PTT model was the constitutive equation derived from network theory. What’s more, it can better describe the rheological properties of polymer melts as well as high shear rates and deformation tensors. The Giesekus model can describe the region where the viscosity of a fluid remains constant at low and high shear rates. The forming process of fibers is mainly influenced by stretching and cooling air, and the crystallization process is actually a combination of static crystallization and stretch-induced crystallization. The process of non-isothermal crystallization is essentially a process of energy conversion. Therefore, the path change of temperature parameters plays a crucial role in the crystallization of fibers. The energy equation was simulated using the material dataset publicly available in Nylon 6. At the same time, there was a maximum temperature difference between the core of the spinneret and the surface, which gradually decreased throughout the process of spinning. The variation throughout the process of spinning is shown in Figure 4.

Figure 4 indicates the predicted temperature distribution along the fiber centerline and free surface. Due to the effect of thermal convection cooling, the surface of fibers and air undergo convective heat transfer. When fluid particles move downstream, the temperature decreases, and the temperature along the surface of fibers is lower than that along the center of fibers. Considering that viscous heat generation is ignored in the calculation, combined with the data values in Figure 4a. The temperature predicted by the PTT model differs from the maximum experimentally measured value by less than 3%. Therefore, PTT shows the best agreement among the three models. At the same time, the PTT model is the constitutive equation that describes the viscoelasticity of the polymer using molecular network models and relaxation time. The model contains two significant parameters, that is, the stretch viscosity control parameter as well as the continuous medium slip control parameter.

By analyzing Figure 4b, it can be observed that there is a significant temperature difference between the center of fibers and the outer surface a certain distance from the extrusion nozzle. Moreover, the temperature difference between the surface of fibers and the center of fibers at a speed of 15 m/s was greater than that at 70 m/s, indicating that the lower the stretching rate, the greater the difference between the fiber surface temperature and the center of temperature.

### 4.2. Input Parameters

In our study, based on the established kinetic model, Nylon 6 BHS and CN9987 resin materials were used as the carrier. Table 1, Table 2 and Table 3, respectively, list the physical as well as rheological parameters, process parameters, and operational parameters of the simulated input materials, corresponding to the parameters in the simulation. These parameters are derived from data reported by researchers [13,15], so these data were cited in our study.

## 5. Results and Discussion

The uneven distribution of melt-spinning fibers in the radial structure is a key factor restricting the development of high-performance, high-value-added fibers. Throughout the entire process of melt spinning, the differences in the radial structure distribution of fibers and the changes in paths of crystallization are the coordinated effects of many parameters. It is difficult to achieve effective control through unilateral parameter control, and it is not possible to discover the regular changes and quantitative value changes in the path. Even existing online detection methods such as infrared detection and high-speed photography can only perform discrete detection on a limited number of fixed positions under the condition of characteristic environments, and the results of detection are not only affected by subjective observations but also greatly interfered by the accuracy of detection technology.

Therefore, the model we established above was used to simulate the process of melt-spinning fiber formation, taking crystallization into account. The joint simulation method was used to predict the temperature, velocity, strain rate tensor, and stress tensor distribution of nylon 6 fibers along different directions. Meanwhile, through the model, we also explored the effects of conditions of spinning, including the inlet flow rate *Q*, winding speed *V*, extrusion temperature *T*, and crystallization of fibers θ. The model is more applicable to “Melt-spinning Methods” and “Viscoelastic Non-Newtonian Fluids”. The specific discussion is as follows.

### 5.1. Research on the Paths of Changes in Speed

The new model broke through the traditional assumption of radial temperature and uniform molecular orientation; introduced free surface thermal convection *B*, and semi-crystalline width $D_{\left(k\right)}^{*}$; and established crystalline and semi-crystalline phases as the Giesekus and PTT constitutive models. At the same time, the Nakamura dynamic equation was introduced in the crystallization model, so the crystallization inflection point was that the appearance was no longer influenced by human intervention but generated in a natural state through established models.

**Figure 5:**
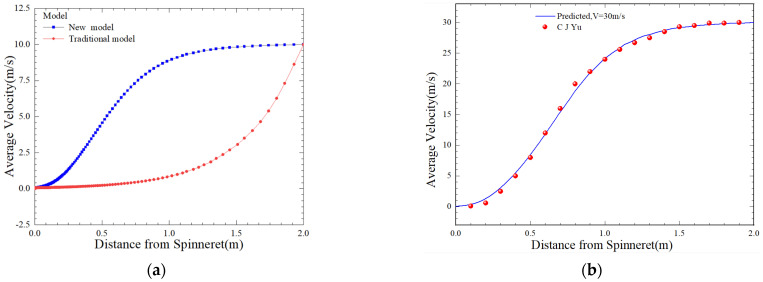
Speed variation throughout the spinning process. (**a**) Contrast of different models [15]; (**b**) contrast of simulation and experiment [37] (*Q* = 3 × 10^−8^ m^3^/s, *T* = 533 k, *T*_0_ = 293 k, *V*_1_ = 10 m/s, *V*_2_ = 30 m/s, *V*_0_ = 0.4 m/s, *L* = 2 m).

To verify the superiority of our model, we compared the predicted speed results of our model with the traditional single-phase model mentioned in the Introduction [15]. Under the same process of parameters, we compared the numerical simulation results of the stretching speed distribution of our model at a winding speed of 30 m/s with the experimental results [37], which were consistent. This indicated that our method simulated the forming process of fibers in a similar manner to the actual spinning and the process of fiber production, providing credibility for the model parameters used in further calculations. The results are shown in Figure 5.

**Figure 6:**
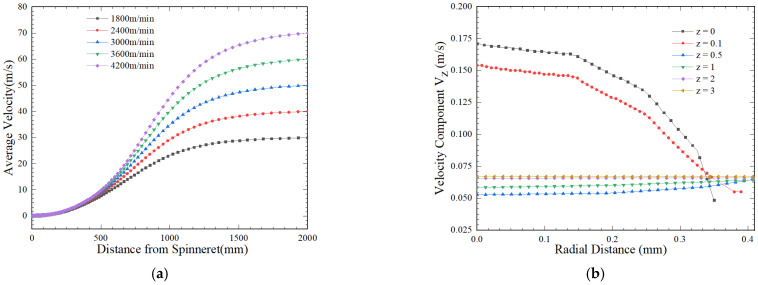
Comparison of velocity distribution at speeds of 30 m/s–70 m/s. (**a**) Axial distribution of take-up velocity along the spinning process; (**b**) radial distribution of velocity at different z points (*Q* = 4.5 × 10^−8^ m^3^/s, *T* = 533 k, *T*_0_ = 293 k, *V*_0_ = 0.4 m/s, *L* = 2 m).

Figure 6 indicates that five speeds represent the changes in high-speed spinning, and it was found that the trend of change in the stretching rate is the same at different stretching rates. However, the magnitude of the change in the stretching rate was variable. When L =1 m, the distribution of stretching rate values is 23.7 m/s, 29.9 m/s, 35.8 m/s, 41.4 m/s, and 46.8 m/s. The faster the winding speed, the more pronounced the S-shaped distribution of the stretching rate of the melt, and there was a region of rapid increase, followed by a stable speed. At the same time, it can be seen from Figure 6b that at a distance of 0.1 mm from the spinneret, the radial velocity component along the z-direction shows a sharp downward trend. However, as the distance from the spinneret reaches 1 mm, the velocity is relatively calm and shows a slow upward trend overall.

### 5.2. Temperature Distribution along the Radial Direction at Different Z Points of the Filament

**Figure 7:**
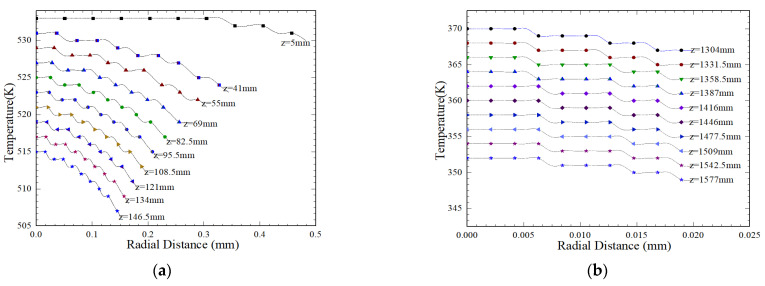
Distribution of radial temperature at different z positions of fibers. (**a**) Initial deformation zone (*z* value 5 mm–150 mm); (**b**) solidification zone (*z* value 1300 mm–1600 mm).

The temperature distribution along the radial direction at different z points under the parameters of the wall temperature *T* = 533 k, stretching rate *V* = 30 m/s, cooling temperature *T*_0_ = 293 K, blowing speed *V*_0_ = 0.4 m/s, and inlet flow rate of 4.5 × 10^−8^ m^3^/s is shown in Figure 7.

Figure 7 indicates the radial temperature distribution at different axial positions in the initial deformation zone and near the solidification zone. As a result of the large temperature difference, the radial temperature distribution bends in the initial deformation zone and becomes gentle near the solidification point. At *z* = 5 mm, the temperature is still close to the extrusion temperature $T_{o}(k)$. As z increases, due to heat transfer between the surrounding medium and the surface of fibers, the temperature decreases and ∂T/∂r increases. At *z* = 1304 mm, the leading order of radial temperature variation developed, and (∂T/∂r)/(∂T/∂z) is 0. Further downstream, for *z* > 1304 mm, this leading order radial variation, ∂T/∂r≫∂T/∂z, is shown in Figure 7b.

### 5.3. The Effects of the Draw-Down Velocities

**Figure 8:**
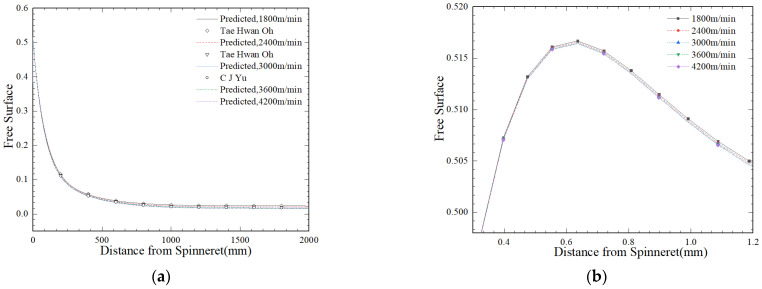
Prediction of fiber surface with different take-up velocities. (**a**) Comparison of the predicted free surface and experimental measurement [13,37]; (**b**) changes in the surface of the filament in the swollen area (*Q* = 4.5 × 10^−8^ m^3^/s, *T* = 533 k, *T*_0_ = 293 k, *V*_0_ = 0.4 m/s, *L* = 2 m).

To investigate the effect of different stretching speeds on the crystallization of microcrystals inside fibers, different winding speeds were applied at the end of the fibers. The results of simulation are shown in Figure 8. As shown in Figure 8, the predicted results of the model are consistent with the experimental measurements throughout the entire spinning process. Near the spinneret hole, significant fiber swelling and necking are observed due to sudden changes in boundary conditions and viscoelastic fluid properties. Under the studied conditions, the fiber diameter reached its maximum value of *R* = 1.032 mm at a distance of *z* = 0.7 mm from the spray hole on the free surface, with a swelling of 0.27 mm. And as the speed increases, the swelling gradually decreases, because the higher the speed, the greater the tensile stress on the fibers.

**Figure 9:**
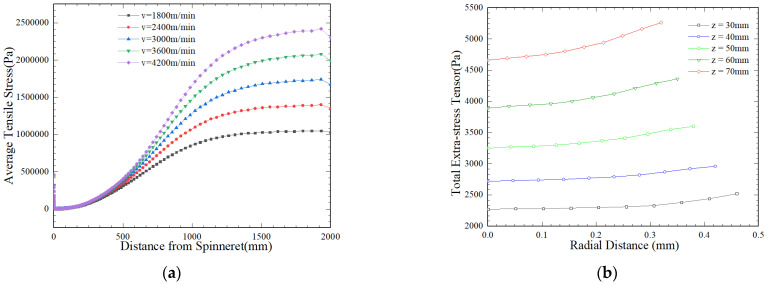

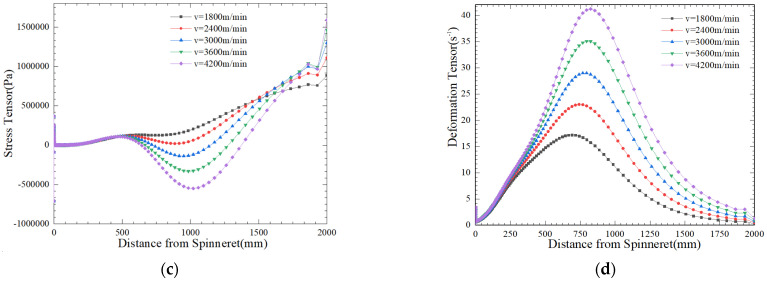
Effect of different take-up velocities on fiber tensor distribution. (**a**) Average stress; (**b**) radial distribution of total external stress tensor at different z points; (**c**) distribution of stress component in the stretching z direction; (**d**) distribution of strain rate tensor along the spinning process.

Figure 9 shows the distribution of the stress tensor and phase transition rate tensor during high-speed spinning in the process of fiber crystallization. As shown in Figure 9a, with an increase in the drafting speed, the average stress on the fibers increases, and the change in stress along the z direction of the spinning process shows an s-shaped trend. *T*_0_ investigate the radial variation in total additional stress in fibers at a stretching speed of *v* = 30 m/s, as shown in Figure 9b, it can be observed that at *z* = 30 mm, the total additional tensor almost followed a straight line along the radial direction, indicating minor variation. However, as the process of spinning increases, at *z* = 70 mm, the total additional tensor shows an almost upward trend along the radial direction, and Figure 9c indicates the variation in stress tensor components along the z direction. Figure 9d shows the variation in the fiber strain rate tensor. As the stretching speed increases, the deformation rate in the neck area becomes sharper with an increase in flow velocity.

**Figure 10:**
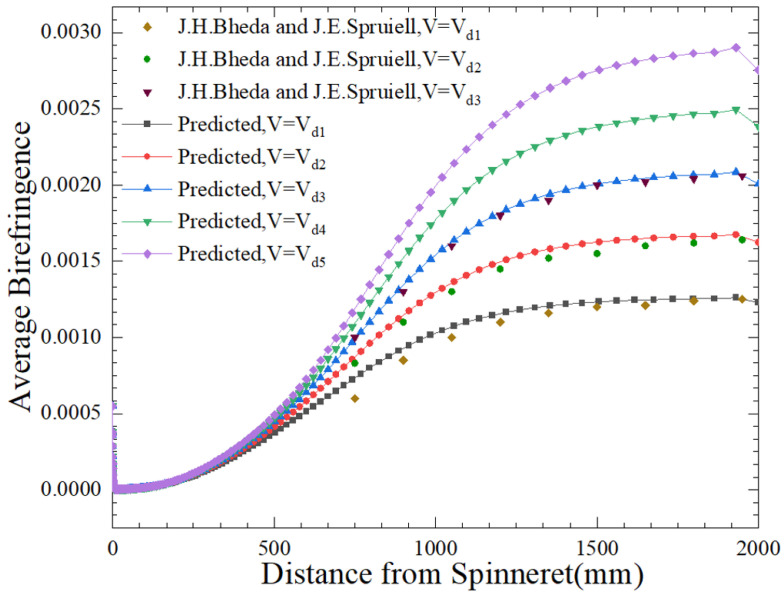
Birefringence distribution of silk slips at different drafts throughout the spinning process [38] (*V*_d1_ = 30 m/s, *V*_d2_ = 40 m/s, *V*_d3_ = 50 m/s, *V*_d4_ = 60 m/s, *V*_d5_ = 70 m/s).

Figure 10 indicates the average birefringence distribution of the predicted fiber cross-sections at different *z* points throughout the process of spinning using numerical solutions. At a given stretching rate, the birefringence index increases with an increase in the spinning distance *z* and tends to reach equilibrium further along the spinning distance. For a given cross-sectional position *z*, the birefringence index increases with an increase in the stretching speed.

What’s more, according to Figure 10, the birefringence experimental data at three different stretching rates of 1800 m/min, 2400 m/min, and 3600 m/min were compared with the values of data simulated by the model established in this study. Firstly, from a qualitative analysis perspective, its overall trend of change remains consistent. Secondly, from a quantitative perspective, the refractive index values of fibers undergoing the same process of spinning are basically consistent. However, the consistency between the predicted values and experimental observations is not as good quantitatively as the temperature changes on the free surface, especially at high stretching rates. The reason for this is due to the lack of elongation viscosity data, and the PTT model parameters are determined by fitting shear viscosity and combining it with shear control parameters. However, the results of previous numerical studies indicate that the predicted fiber surface matches well with the experimental observations, which provides credibility for the calculation of the model. Meanwhile, the speed variation throughout the process of spinning is highly consistent with the experimental results. Combining Equations (41) and (44), birefringence is a function of temperature and stretching, and crystallization is a function of birefringence. This indirectly proves the correctness and accuracy of the model of crystallization kinetics established in this study.

**Figure 11:**
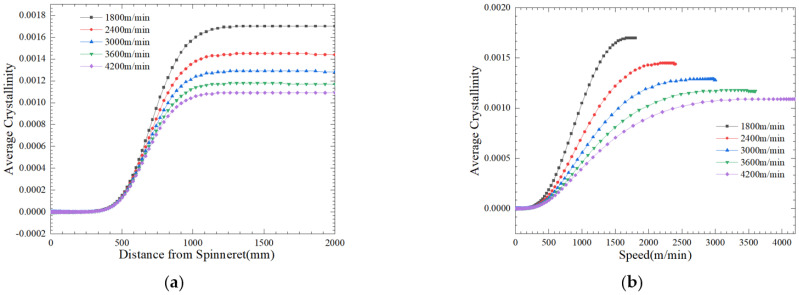

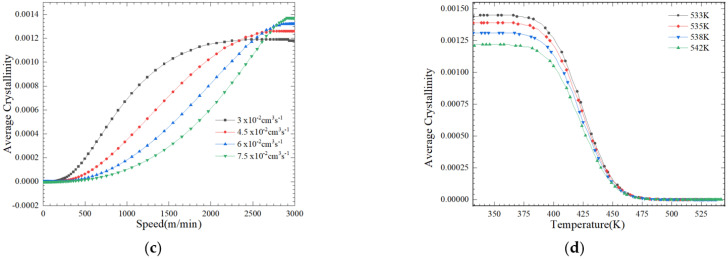
Effect of different spinning conditions on fiber crystallinity. (**a**) Different take-up velocities; (**b**) relationship between take-up velocities and crystallinity; (**c**) inlet flow rate; (**d**) spinneret wall temperature.

Figure 11a shows the average crystallinity of the fiber cross-section throughout the process of spinning at different drafting speeds. Under the given process conditions, an increase in crystallinity with an increase in the process of spinning *z* ultimately reached equilibrium. The position of the equilibrium point is related to the magnitude of the stretching speed. With an increase in the stretching speed, the relative residence time of macromolecules inside the fibers will decrease, and the crystal nucleus will not have enough growth time, causing crystallization to migrate from upstream to downstream. Figure 11b shows the variation pattern of stretching speed and final crystallinity. Under the same process parameters, the higher the stretching speed, the greater the tensile stress on the fibers, and the crystallization rate is higher. On the contrary, due to the influence of tensile force, an increase in stretching speed will result in a decrease in the final fiber crystallinity. Figure 11c shows the effect of different spinning flow rates on the process of fiber crystallization. It can be observed that as the inlet flow rate increases, the crystallization rate of fibers slows down in the initial stage, but the final crystallinity of fibers increases. Figure 11d shows the effect of wall temperature on the crystallization of fibers. As the wall-melting temperature increases, the crystallization rate actually decreases, and the crystallinity of the resulting fibers also decreases.

**Figure 12:**
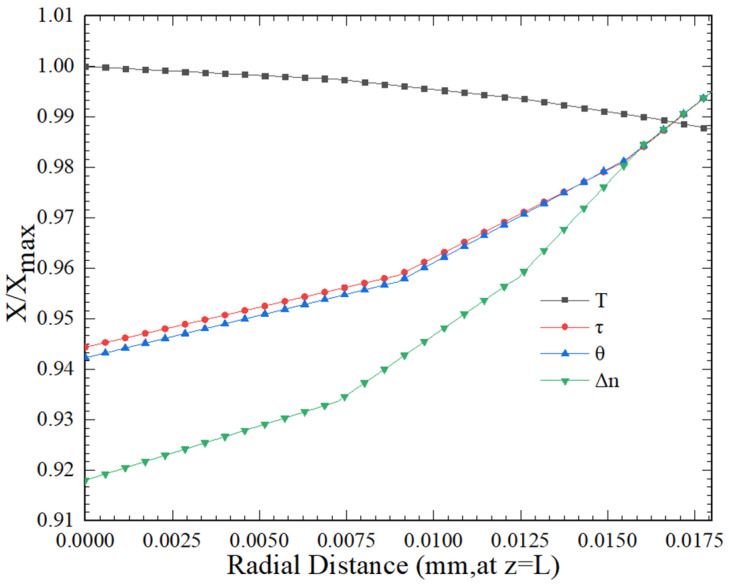
Distribution of radial temperature *T*, stress tensor τ, crystallinity θ, and birefringence Δn ( $X_{\max}$ represents the maximum value of *X*).

Figure 12 summarizes the effect of $X/X_{\max}$ on the radial distribution of fibers. *X* represents temperature *T*, stress tensor τ, crystallinity θ, and birefringence Δn. To define ${\left(X/X_{\max}\right)}_{\min}$ as the minimum value of $X/X_{\max}$, ΔX can be regarded as the quantitative analysis value for evaluating the non-uniformity of *X*, and the calculation expression is shown in (54).

(54) $$\Delta X=1-(\frac{X}{X_{\max}}{)}_{\min}\;\mathrm{at}\;z=L$$

If the value of ΔX is 0, it represents a uniform distribution, and if the value of ΔX is 1, it represents 100% relative variation. Regardless of the temperature, stress tensor, birefringence index, and crystallinity, the changes $X/X_{\max}$ intersect at a point of radial *z* = 0.016 mm. This intersection indicates that the fluid non-uniformity of the fibers during formation is minimized at this point, and the system of formation reaches the most stable state. Before 0.016 mm, the temperature *T* exhibits the smallest non-uniformity change. After the intersection point, these non-uniformities increase with an increase in the stretching rate, which has a significant impact on the fiber microstructure. This requires special consideration in fiber preparation and the process of control.

## 6. Conclusions

Through the two-phase model established in this research, which included changes in the fiber radial structure and microcrystalline crystallization in melt spinning, the change paths of the process of parameters related to the entire spinning process and fiber mechanical properties can be obtained. Compared with traditional models, firstly, the innovation of the model established in this study is the specific distinction between amorphous and semi-crystalline phases in the rheological response of the system. In addition, another key innovation of the model is that the emergence of crystallization inflection points is no longer a result of human intervention but, rather, occurs in a natural state, which is not present in traditional models. Finally, our model can accurately predict the changes in variable parameters related to crystallization, such as stress tensor, strain rate tensor, and crystallization rate, under different stretching rates. Understanding the path changes of these parameters can provide important guidance and optimization for the future industrial preparation of high-performance fibers.

The model was modified and validated through nylon 6 BHS resin and CN9987 resin spinning simulation and experimental results, and the final results showed that the model exhibited strong robustness across a wide range of spinning conditions and parameter processes. The model successfully predicted the axial and radial variations in the wire temperature, velocity, strain rate tensor, birefringence index, and stress tensor under different drafting conditions. What’s more, the influence of spinning conditions on the fiber crystallization process was studied through model simulation. The results indicated that along with an increase in the inlet flow rate, the crystallinity of the fiber increased, while with an increase in the winding speed, the crystallinity of the fiber decreased. At the same time, the wall of the spinneret hole also had an impact on the fiber crystallization, with an increase in the temperature and a decrease in the final fiber crystallinity.

## Figures and Tables

**Table 1:** Geometry and model parameters for dynamic equations.

Processing Parameter	Value
Diameter of entrance channel, *D*_0_ (mm)	0.76
Lenth of exit channel, *L*_0_ (mm)	2.42
Distance from spinneret, *L* (mm)	2 × 10^3^
Extrusion temperature, *T*_0_ (k)	533
Molten temperature, *T*_m_ (k)	503
Maximum rate temperature, *T*_max_ (k)	419
Cooling temperature, *T*_c_ (k)	298
Blowing speed, *V*_air_ (m/s)	0.4

**Table 2:** Physical and rheological parameters.

Properties	Parameters	References
Viscosity, η (Pa·s)	750	[14]
Relaxation time, λ (s)	0.015	[31]
Elongational parameter, ε	0.2	[31]
Mobility factor, ξ	0.001	[31]
Additional viscosity, (Pa·s)	0.25	[31]
Density, ρ (kg/m^3^)	973	[15]
Thermal conductivity, k (w/m/k)	0.2	[31]
Heat capacity, *C* (J/kg/k)	1.3497 × *T* + 1982.76	[31]
Arrhenius, α (k)	4470	[31]
Ultimate crystallinity, $\theta_{\infty }$	0.45	[31]
Maximum crystallization rate at *T*_max_, *K*_max_ (s^−1^)	0.14	[31]
Crystallization half width, $D_{\left(k\right)}^{*}$	46	[15]
Stress induced coefficient, *c*	225	[15]
Stress-optical coefficient, $C_{\mathrm{op}}^{*}$ (m^2^N^−1^)	1.25 × 10^−9^	[15]
Amorphous intrinsic birefringence, $\Delta \alpha_{i}$	8.25 × 10^−2^	[31]
Heat of fusion, *H*_f_ (Jkg^−1^)	1.884 × 10^5^	[15]

**Table 3:** Conditions of melt spinning in the simulation.

Number	Volume Flow Rate (mm^3^/s Hole)	Spinning Temperature (k)	Circular Air Temperature (k)	Spinning Speed (m/min)
1	30	533	293	3000
2	45	533	293	3000
3	60	533	293	3000
4	75	533	293	3000
5	60	533	293	1800
6	60	533	293	2400
7	60	533	293	3600
8	60	533	293	4200
9	60	535	293	3000
10	60	538	293	3000
11	60	542	293	3000
12	60	545	293	3000
13	60	533	298	3000
14	60	533	300	3000
15	60	533	304	3000

## Data Availability

The original contributions presented in the study are included in the article, further inquiries can be directed to the corresponding author.
